# Television Viewing from Young Adulthood to Middle Age and Premature Cardiovascular Disease Events: A Prospective Cohort Study

**DOI:** 10.1007/s11606-024-08951-z

**Published:** 2024-08-22

**Authors:** Jason M. Nagata, Eric Vittinghoff, Chloe M. Cheng, Erin E. Dooley, Feng Lin, Jamal S. Rana, Stephen Sidney, Cora E. Lewis, Kelley Pettee Gabriel

**Affiliations:** 1 Division of Adolescent and Young Adult Medicine, Department of Pediatrics, University of California, San Francisco, San Francisco, CA USA; 2 Department of Epidemiology and Biostatistics, University of California, San Francisco, San Francisco, CA USA; 3 Department of Epidemiology, University of Alabama at Birmingham, Birmingham, AL USA; 4 Division of Cardiology, Kaiser Permanente Northern California, Oakland, CA USA; 5 Division of Research, Kaiser Permanente Northern California, Oakland, CA USA

**Keywords:** screen time, television, sedentary behavior, cardiovascular disease, atherosclerotic disease, coronary heart disease, myocardial infarction, stroke, heart failure, young adults

## Abstract

**Background:**

Previous literature has explored the relationship between television viewing and cardiovascular disease (CVD) in adults; however, there remains a paucity of longitudinal data describing how young adult television viewing relates to premature CVD events.

**Objective:**

To ascertain the relationship between level and annualized changes in television viewing from young adulthood to middle age and the incidence of premature CVD events before age 60.

**Design:**

The Coronary Artery Risk Development in Young Adults (CARDIA) study, a prospective community-based cohort with over 30 years of follow-up (1985–present).

**Participants:**

Black and White men and women who were 18–30 years old at baseline (1985–1986).

**Main Measures:**

Independent variables: Individualized television viewing trajectories were developed using linear mixed models. Dependent variables: Fatal and nonfatal coronary heart disease (CHD), heart failure, and stroke outcomes were analyzed separately and as a combined CVD event outcome.

**Key Results:**

Among 4318 included participants, every 1-h increase in daily hours of television viewing at age 23 was associated with higher odds of incident CHD (adjusted odds ratio [AOR] 1.26, 95% confidence interval [CI] 1.06–1.49) and incident CVD events (AOR 1.16, 95% CI 1.03–1.32). Each additional hour of daily television viewing annually was associated with higher annual odds of CHD incidence (AOR 1.55, 95% CI 1.06–2.25), stroke incidence (AOR 1.58, 95% CI 1.02–2.46), and CVD incidence (AOR 1.32, 95% CI 1.03–1.69). Race and sex modified the association between television viewing level at age 23 and CHD, heart failure, and stroke, with White men most consistently having significant associations.

**Conclusions:**

In this prospective cohort study, greater television viewing in young adulthood and annual increases in television viewing across midlife were associated with incident premature CVD events, particularly CHD. Young adulthood as well as behaviors across midlife may be important periods to promote healthy television viewing behavior patterns.

**Supplementary Information:**

The online version contains supplementary material available at 10.1007/s11606-024-08951-z.

## INTRODUCTION

Cardiovascular disease (CVD) is the leading cause of death in the United States (US), with >130 million US adults (45.1%) projected to have CVD by 2035.^1^ Young adulthood represents an under-researched developmental period when individuals develop CVD risk factors and lifestyle behaviors that persist throughout adulthood.^2,3^

Television viewing is the most common leisure-time sedentary behavior and screen modality despite the advent of contemporary screen modalities.^4^ Criterion validity of self-reported television viewing estimates tends to be stronger than those for total sitting time estimates given programming is often structured in 30-min increments, allowing for easier recall.^5,6^ As such, television viewing has been extensively used in prior research as a proxy measure for sedentary behavior. Sedentary behavior is distinct from a lack of physical activity and is defined as “any waking behavior characterized by an energy expenditure <1.5 metabolic equivalents (METs), while in a sitting, reclining or lying posture.”^7^ Television viewing is a type of sedentary behavior that is most often done sitting, which is thought to have physiological adaptations that are distinct from standing.^8^ An individual who runs for 60 min daily but also sits 4 h per day in front of a television is considered both physically active and sedentary.^9^ Several studies have demonstrated that independent of physical activity, increased television viewing time is associated with increased risk for CVD risk factors (e.g., hypertension, diabetes, dyslipidemia)^10,11^ and events (e.g., coronary heart disease [CHD], heart failure, stroke).^12–16^

Meta-analyses of prospective cohort studies have shown that prolonged television viewing time may increase the risk of all-cause mortality and CVD outcomes.^17,18^ Further, a recent systematic review and meta-analysis of 24 prospective cohort studies suggested that sedentary behavior/television viewing increases cardiovascular and mortality risk in a dose-dependent manner.^19^ Data from the Coronary Artery Risk Development in Young Adults (CARDIA) database found that increased television viewing in young adulthood was associated with CVD risk factors including hypertension, diabetes, high triglycerides, and obesity; the present study will expand upon these findings by investigating associations between television viewing and CVD events.^11^

Though previous studies have shown associations between television viewing time and the incidence of CVD events, these studies have largely been conducted in middle-aged and older adults at baseline, with relatively short follow-up periods. The 2018 Department of Health and Human Services (HHS) *Physical Activity Guidelines for Americans* identified important evidence gaps in the literature about sedentary behavior and associations with CVD events, especially those related to young adulthood. Current guidelines acknowledge that young adults may have unique growth and developmental considerations, but group all adults aged 18–65 years together, citing insufficient evidence in the young adult age group.^20^ The *Guidelines* also noted that there was insufficient evidence to determine whether the relationship between sedentary behavior and incident CVD events varied by race or sex.^20^

Furthermore, little is known about how television viewing trajectories (patterns over age and time) from young adulthood to middle age influence CVD events. Young adulthood may set television viewing trajectories for the rest of adulthood and therefore, may represent an important window for early intervention.^21,22^

Using data from CARDIA—an ongoing population-based prospective cohort study starting in young adulthood—this study aimed to determine the association between television viewing trajectories (level in young adulthood and slope through the adult lifespan to midlife) and incidence of premature CVD events (e.g., CHD, heart failure, stroke) before age 60. In addition, the study investigated whether race or sex modified the relationship between television viewing level and change throughout the adult life course and the incidence of premature CVD events.

## METHODS

### Study Population

Young adults (*N* = 5115) who self-identified as Black or White race were enrolled in CARDIA in 1985–1986 from four urban sites (Birmingham, AL; Chicago, IL; Minneapolis, MN; and Oakland, CA). The cohort was structured at baseline to be approximately balanced within each center by age (18–24 years and 25–30 years), sex (male and female), self-identified race (Black and White), and level of education (high school or less and higher than high school). One participant later withdrew consent and was excluded after the baseline examination. Overall, the cohort exhibited high retention rates—86%, 81%, 77%, 74%, 72%, 72%, and 71% at follow-up examinations in years 5, 7, 10, 15, 20, 25, and 30, respectively. The year 5 follow-up exam (cohort aged 23–35 years) is the present study’s baseline as it was the first exam during which television viewing was assessed, with *N* = 4318 with television viewing data. Further information about the study design is detailed elsewhere.^23^ Study procedures were approved by each site’s institutional review board and all participants provided written informed consent.

### Measures

#### Exposure Variable: Television Viewing

At the follow-up exams in years 5, 7, 10, 15, 20, 25, and 30, television viewing was assessed using the interviewer-administered CARDIA Physical Activity History Questionnaire.^24^ The questionnaire asks, “During leisure time do you watch television or other video programming?” Responses were one of the following: never, seldom, sometimes, often, or very often. If participants indicated any response other than “never” (e.g., seldom, sometimes, often, or very often), they were asked, “On the average, about how many hours per day do you watch television [or other video programming]?” The reported number (or 0 for “never”) was used to compute average daily viewing hours. Television viewing measures based on self-report have shown a high level of agreement (95% of values within 4 h of the mean) with objective measures (e.g., electronic television monitor) and a significant moderate positive correlation (Spearman’s *p* = 0.54, *p* < 0.001).^25^ Television watching (hours per week) based on self-report has demonstrated acceptable test-retest reliability (7-day test-retest intraclass correlations 0.76–0.81).^26,27^

#### Outcome Variables: CVD Events

Participants were assessed for potential outcomes through annual participant contact.^28,29^ Vital status was assessed every 6 months and searches were performed at least annually for participants who could not be contacted, using resources such as the US Social Security Death Index, obituary search engines, and/or close contacts. National Death Index searches were conducted approximately every 5 years. Medical records, death certificates, informant interviews, and autopsy reports were requested, depending on the outcome. Outcomes included coronary heart disease (myocardial infarction, non-myocardial infarction acute coronary syndrome), heart failure (congestive heart failure, other non-atherosclerotic cardiac disease), and stroke (stroke, not including transient ischemic attacks). A combined CVD events variable included myocardial infarction, non-myocardial infarction acute coronary syndrome, congestive heart failure, stroke, carotid artery disease, peripheral artery disease, death due to atherosclerotic coronary heart disease, other non-atherosclerotic cardiac disease, and atherosclerotic disease other than coronary or stroke. Each record was reviewed by two members of the CARDIA Endpoints Surveillance and Adjudication Subcommittee (ESAS), who applied standard outcome definitions to categorize events. The full CARDIA ESAS Committee resolved disagreements. Data on CVD events were collected through August 31, 2020. Approximately 85% of participants had been contacted in the 2 years, and 91% in the 5 years, prior to August 31, 2020.

##### Covariates

Standardized questionnaires were used to obtain self-reported data on age (years), sex (male or female), race (Black or White), level of education (the highest grade of school completed), and a family history of cardiovascular disease (yes or no) at baseline, as well as alcohol use (mL of alcohol consumed per day), smoking status (never, former, or current smoker), and physical activity (continuous exercise units from the CARDIA Physical Activity History Questionnaire^30^ which incorporates frequency and intensity) at each examination.^24,31^ Body mass index (BMI) was calculated with height and weight measurements taken at each examination. Previous literature identified these variables as possible confounders for the association between television viewing and CVD events.^30,32^

#### Statistical Analysis: Summarizing Television Viewing

We modeled television viewing trajectories using a linear mixed model (LMM) for repeated measures to produce concise summaries of television viewing trends over time.^30,32^ Using all observations of daily hours of television viewing before CVD event onset (so that the exposure variables would temporally precede the outcome), we estimated the television viewing slopes, with the aim of utilizing as much data as possible for each participant (i.e., if data from one or more follow-up exam(s) were absent, then data from the remaining available follow-up exams were used) and stabilizing the best linear objective predictions. The LMM had random effects for participant and continuous age, with unstructured covariance, and fixed effects for a four-level joint categorization of sex and race, continuous age, and their interactions. Expected hours of television viewing per day at age 23 (the youngest age of study participants at the year 5 follow-up exam) and the yearly change for each participant were computed using the random and fixed estimates provided by the model.

##### Modeling the Association of Television Viewing with Incident CVD Events

We used Kaplan-Meier methods to estimate the unadjusted cumulative incidence of CVD events (CHD, heart failure, stroke, combined CVD events) by sex and race. The data for each participant were then expanded to include a record for each age between study entry and either the date the CVD event occurred, at censoring by the end of the study, or loss to follow-up. Pooled logistic models were used to estimate the independent associations of the expected hours of television viewing per day at age 23 and the subsequent annual change in television viewing (joint exposure variables) with the onset of CVD events (outcome variables). Each outcome was analyzed with model 1, which adjusted for age only, and model 2, which adjusted for age, race, sex, education, family history of CVD, smoking status, alcohol use, BMI, and physical activity, which have been adjusted for in previous analyses examining the link between television viewing and cardiometabolic disease.^30,32^ A cubic spline in the current age was used in both models 1 and 2 for CVD events. Physical activity, smoking status, alcohol use, and BMI were considered time-varying covariates (i.e., data from each year of the follow-up were used for these). If a covariate was missing data at a specific follow-up exam, the last observation was carried forward. After the primary analysis, we further assessed whether sex and race altered the association between television viewing (level and change) and incident CVD events by testing interaction terms. All analyses were conducted with Stata 17.0 (StataCorp, College Station, TX).

## RESULTS

The demographic and health characteristics of the 4318 participants included in this study (48.8% Black, 51.2% White, and 45.1% male, 54.9% female) are found in Table 1. By the end of the study period, the number of respective events included CHD (*n* = 81), heart failure (*n* = 50), stroke (*n* = 56), or any CVD event (*n* = 171, Table 2). Supplemental Figs. A-D show the cumulative incidence of CVD event outcomes by race and sex.

**Table 1:** Baseline Demographic and Health Characteristics of Participants in the Coronary Artery Risk Development in Young Adults (CARDIA) Study

***N***	**Total**	**White women**	**Black women**	**White men**	**Black men**	***p*-value**
	**4318**	**1165**	**1205**	**1046**	**902**	
**Baseline demographic characteristics**	**Median (IQR)/*n* (%)**	**Median (IQR)/*n* (%)**	**Median (IQR)/*n* (%)**	**Median (IQR)/*n* (%)**	**Median (IQR)/*n* (%)**	
Age (years)	30.0 (27.0 to 33.0)	31.0 (28.0 to 33.0)	30.0 (26.0 to 33.0)	31.0 (28.0 to 33.0)	29.0 (26.0 to 33.0)	<0.001
Highest grade of school completed	14.0 (12.0 to 16.0)	16.0 (13.0 to 16.0)	14.0 (12.0 to 15.0)	16.0 (13.0 to 16.0)	13.0 (12.0 to 15.0)	<0.001
Body mass index (BMI)	24.9 (22.2 to 28.6)	23.0 (21.0 to 26.1)	26.7 (22.7 to 32.3)	24.9 (22.8 to 27.6)	25.5 (23.0 to 28.8)	<0.001
<25 kg/m^2^	2,221 (51.4%)	792 (68.0%)	480 (39.8%)	541 (51.7%)	408 (45.2%)	
25 to 30 kg/m^2^	1,253 (29.0%)	235 (20.2%)	330 (27.4%)	375 (35.9%)	313 (34.7%)	
>30 kg/m^2^	844 (19.5%)	138 (11.8%)	395 (32.8%)	130 (12.4%)	181 (20.1%)	
Family history of cardiovascular disease	861 (19.9%)	220 (18.9%)	255 (21.2%)	198 (18.9%)	188 (20.8%)	0.38
Smoking status						<0.001
Never	2,468 (57.2%)	644 (55.3%)	717 (59.5%)	623 (59.6%)	484 (53.7%)	
Former	610 (14.1%)	265 (22.7%)	101 (8.4%)	165 (15.8%)	79 (8.8%)	
Current	1,240 (28.7%)	256 (22.0%)	387 (32.1%)	258 (24.7%)	339 (37.6%)	
Alcohol (mL of alcohol consumed per day)	2.4 (0.0 to 14.3)	2.4 (0.0 to 9.5)	0.0 (0.0 to 5.1)	7.5 (0.0 to 20.8)	7.2 (0.0 to 25.2)	<0.001
Total physical activity score at enrollment (EU)^a^	311.0 (161.0 to 532.0)	288.0 (158.0 to 468.0)	210.0 (92.0 to 372.0)	415.0 (236.0 to 629.0)	405.5 (223.0 to 672.0)	<0.001
Television viewing						
Total television viewing at exam year 5 (hours per day)	2.0 (1.0 to 3.0)	1.0 (1.0 to 2.0)	3.0 (2.0 to 4.0)	2.0 (1.0 to 2.0)	3.0 (2.0 to 4.0)	<0.001
Estimated total television viewing at age 23 (hours per day)	2.9 (2.3 to 3.8)	2.3 (2.0 to 2.8)	3.6 (2.9 to 4.4)	2.4 (2.1 to 3.0)	3.9 (3.2 to 4.8)	<0.001
Annual increase in television viewing (hours per day)	0.2 (−0.2 to 0.5)	0.3 (−0.1 to 0.7)	0.1 (−0.2 to 0.5)	0.2 (−0.0 to 0.6)	0.0 (−0.3 to 0.3)	<0.001

*IQR*, interquartile range; *EU*, exercise units

^a^A total physical activity score of 300 exercise units (EU) approximates the Health and Human Services recommendations of approximately 150 min of moderate-intensity activity per week

**Table 2:** Associations Between Television Viewing Trajectories and Incidence of Premature Cardiovascular Disease (CVD) Events in the CARDIA Study

	**Number of events among 4318 participants**	**Model 1 (adjusted for age)**^**a**^			**Model 2 (fully adjusted)**^**b**^		
		**OR**	**95% CI**	***p***	**OR**	**95% CI**	***p***
Coronary heart disease (CHD)—fatal or nonfatal (myocardial infarction, non-myocardial infarction acute coronary syndrome)							
Estimated television viewing at age 23 (hours per day)	81	**1.31**	**1.17, 1.46**	**<0.001**	**1.26**	**1.06, 1.49**	**0.01**
Annual increase in television viewing (hours per day)		**1.58**	**1.12, 2.23**	**0.01**	**1.55**	**1.06, 2.25**	**0.02**
Heart failure—fatal or nonfatal (congestive heart failure, other non-atherosclerotic cardiac disease)							
Estimated television viewing at age 23 (hours per day)	50	**1.6**	**1.44, 1.76**	**<0.001**	1.24	1.01, 1.54	0.04
Annual increase in television viewing (hours per day)		1.35	0.91, 2.01	0.14	1.52	1.02, 2.27	0.04
Stroke—fatal or nonfatal (stroke, not including transient ischemic attacks)							
Estimated television viewing at age 23 (hours per day)	56	**1.46**	**1.28, 1.67**	**<0.001**	1.15	0.89, 1.48	0.28
Annual increase in television viewing (hours per day)		**1.64**	**1.02, 2.65**	**0.04**	**1.58**	**1.02, 2.46**	**0.04**
CVD—fatal or nonfatal (myocardial infarction, non-myocardial infarction acute coronary syndrome, congestive heart failure, stroke, carotid artery disease, peripheral artery disease, other atherosclerotic disease other than coronary or stroke)							
Estimated television viewing at age 23 (hours per day)	171^c^	**1.42**	**1.32, 1.53**	**<0.001**	**1.16**	**1.03, 1.32**	**0.02**
Annual increase in television viewing (hours per day)		**1.35**	**1.06, 1.72**	**0.01**	**1.32**	**1.03, 1.69**	**0.03**

Boldface indicates statistical significance (*p* < 0.05)

^a^Model 1 includes estimated television viewing level at age 23, annual increase in television viewing, age. Separate models are presented for each outcome (CHD, heart failure, stroke, CVD)

^b^Model 2 includes estimated television viewing level at age 23, annual increase in television viewing, age, race, sex, education, family history of CVD, smoking status, alcohol, body mass index, and physical activity. Smoking, alcohol, body mass index, and physical activity were time-varying. Separate models are presented for each outcome (CHD, heart failure, stroke, CVD)

^c^Number of CVD events is smaller than the total of CHD, heart failure, and stroke as some individual participants had multiple types of CVD events

Pooled logistic regression model estimates for the associations between CVD event incidence and the two television viewing summaries (daily hours of television viewing at age 23 and subsequent increases in daily hours of television viewing) are shown in Table 2. In the fully adjusted model (model 2), every additional hour of television viewing at age 23 was associated with higher odds of incident CHD (adjusted odds ratio [AOR] 1.26, 95% confidence interval [CI] 1.06–1.49) and incident CVD events (AOR 1.16, 95% CI 1.03–1.32). Each additional hour increase of daily television viewing annually was associated with higher annual odds of CHD incidence (AOR 1.55, 95% CI 1.06–2.25), stroke incidence (AOR 1.58, 95% CI 1.02–2.46), and CVD incidence (AOR 1.32, 95% CI 1.03–1.69). In the fully adjusted model, neither television viewing at age 23 nor annual change was associated with incident heart failure.

Sex and race categories modified the effect of television viewing level at age 23 on incident CHD, heart failure, and stroke (all *p* < 0.05), but not the combined CVD events outcome. In analyses stratified by race and sex (Table 3), television viewing level at age 23 was most strongly associated with odds of subsequent CHD among White women (AOR 1.85, 95% CI 1.26–2.72) and White men (AOR 1.50, 95% CI 1.28–1.76) but not among Black women or men. Television viewing level at age 23 was most associated with heart failure (AOR 2.13, 95% CI 1.60–2.84) and stroke (AOR 2.48, 95% CI 1.92–3.22) among White men but not in other subpopulations. Sex and race categories did not significantly modify the effect of annual increases in television viewing levels on incident CHD, heart failure, stroke, or CVD events (all *p* > 0.05).

**Table 3:** Associations Between Television Viewing Trajectories and Incidence of Premature Cardiovascular Disease (CVD) Events in the CARDIA Study, Stratified by Race and Sex

	**Estimated television viewing at age 23 (hours per day)**			**Annual increase in television viewing (hours per day)**		
	**OR**^**a**^	**95% CI**	***p***	**OR**^**a**^	**95% CI**	***p***
Coronary heart disease (CHD)—fatal or nonfatal (myocardial infarction, non-myocardial infarction acute coronary syndrome)						
			0.016^b^			0.54^b^
White women	1.85	1.26, 2.72	0.002	1.12	0.49, 2.55	0.79
Black women	1.10	0.78, 1.54	0.60	2.18	1.20, 3.95	0.01
White men	1.50	1.28, 1.76	<0.001	1.40	0.82, 2.39	0.22
Black men	1.00	0.76, 1.32	1.00	1.32	0.57, 3.06	0.51
Heart failure—fatal or nonfatal (congestive heart failure, other non-atherosclerotic cardiac disease)						
			0.024^b^			0.10^b^
White women	1.48	0.74, 2.94	0.27	1.23	0.22, 6.94	0.81
Black women	1.12	0.80, 1.57	0.50	1.37	0.75, 2.50	0.31
White men	2.13	1.60, 2.84	<0.001	2.98	2.04, 4.35	<0.001
Black men	1.30	1.04, 1.62	0.02	1.69	0.90, 3.16	0.10
Stroke—fatal or nonfatal (stroke, not including transient ischemic attacks)						
			<0.001^b^			0.31^b^
White women	0.20	0.04, 1.17	0.07	0.92	0.14, 6.20	0.93
Black women	0.99	0.76, 1.30	0.97	1.78	1.04, 3.03	0.034
White men	2.48	1.92, 3.22	<0.001	2.53	1.23, 5.22	0.012
Black men	1.19	0.81, 1.76	0.37	0.96	0.44, 2.10	0.92
CVD—fatal or nonfatal (myocardial infarction, non-myocardial infarction acute coronary syndrome, congestive heart failure, stroke, carotid artery disease, peripheral artery disease, other atherosclerotic disease other than coronary or stroke)						
			0.06^b^			0.80^b^
White women	1.41	0.91, 2.19	0.13	1.06	0.50, 2.27	0.88
Black women	1.03	0.86, 1.23	0.74	1.33	0.90, 1.97	0.16
White men	1.62	1.20, 2.20	0.002	1.60	1.00, 2.57	0.051
Black men	1.21	1.00, 1.45	0.05	1.26	0.81, 1.95	0.30

^a^Models include estimated television viewing level at age 23, annual increase in television viewing, age, education, family history of cardiovascular disease, smoking status, alcohol, body mass index, and physical activity. Smoking, alcohol, body mass index, and physical activity were time-varying

^b^Tests for interactions with race/ethnicity and sex. *p* for interaction listed above stratified outcomes

## DISCUSSION

In this prospective cohort study characterizing the young adult to midlife transition, we found that after adjustment, higher television viewing levels during young adulthood were associated with incident premature CHD and CVD events, independent of television viewing later in adulthood. Therefore, young adulthood may be an important window for intervention in television viewing habits to prevent adverse cardiovascular outcomes. In addition, for any given young adult television viewing set-point, an increase in television viewing through midlife was associated with incident premature CHD, stroke, and CVD events. Thus, it may be important to reduce television viewing levels throughout the adult life course.

Our findings are consistent with previous studies showing associations between television viewing levels and subsequent CHD events^16,33^ and CVD events.^12,13,15^ However, while previous studies have shown no association between television viewing levels and incident stroke,^34,35^ our study found that an increase in television viewing throughout adulthood was associated with incident stroke. Such findings may indicate that increases in television viewing over time may be more important for the risk of incident stroke than television viewing levels in young adulthood.

Our study adds to knowledge in the field by investigating both estimated television viewing levels at age 23 and television viewing trajectories with follow-up from young to middle adulthood, whereas most previous studies have examined middle-aged or older adults at baseline, had shorter follow-up periods, and/or studied television viewing levels at a single time point rather than trajectories over time.

Our study also found significant race and sex-stratified associations between television viewing levels and CVD outcomes, adding to the HHS-reported evidence gap on whether the association between television viewing level and incident CVD events varies by race or sex.^20^ However, it should be noted that there were relatively few events overall, and race-sex stratified analyses may be statistically underpowered. Further research should attempt to replicate our race and sex-stratified results and elucidate possible mechanisms underlying differences in outcomes.

Several mechanisms may explain the overall relationship between television viewing and an increase in incident premature CVD events. Sedentary behavior is a risk factor for CVD.^36–38^ Television viewing is primarily sedentary and may displace time that could otherwise be spent physically active, which generally decreases the risk of CVD. However, our study showed associations even after adjusting for physical activity. Another theory is that sedentary behavior has unique physiological effects and metabolic health consequences separate from those of low physical activity.^8^ It is also possible that television viewing leads to greater caloric consumption^39^ and consumption of unhealthy foods,^40^ which then increases cardiometabolic risk (e.g., hypertension, diabetes, dyslipidemia).^11,41,42^ Such eating habits may be explained by distracted eating, eating despite the absence of hunger cues while watching television, and increased exposure to food advertisements on television.^43,44^ Future studies should continue to evaluate and further clarify the potential mechanisms underlying the association between television viewing levels in young adulthood and trajectory over the adult life span and incident premature CVD events.

### Clinical Perspectives

The present study’s findings are potentially significant for public policy, public health, and clinical practice. Our research helps address evidence gaps identified by the 2018 HHS Physical Activity Guidelines for Americans Scientific Report—specifically, evidence regarding associations between sedentary behavior and incident CVD outcomes in young adulthood and differences in associations by race and sex.^20^

Clinicians can consider inquiring about television viewing habits and other sedentary behaviors, especially in young adulthood. In addition, clinicians can encourage physical activity, especially during leisure time. Our research further informs strategies for CVD outcome prevention, suggesting that it is especially important to start preventive interventions in young adulthood, as emphasized by our finding that higher television viewing levels in young adulthood may be related to a greater risk of premature CVD events.

### Limitations and Strengths

The limitations and strengths of this study should be noted. In this study, television viewing was the only form of screen exposure examined, as it was the primary form of screen exposure when first assessed (1990–1991 and throughout most of the study).^45^ Future research should investigate contemporary screen modalities (e.g., mobile phones, computers, and tablets) and CVD outcomes. Further, the measurement of television viewing in our study was based on self-report, which is subject to recall bias and social desirability bias, though the latter would underestimate television viewing and bias towards the null hypothesis. An increase of an hour per day of television viewing annually is large, so effect sizes are smaller for smaller changes in television viewing over time. In addition, CARDIA was designed to study Black and White young adults, which may limit generalizability to other races/ethnicities. Of note, Bureau of Labor Statistics data indicates that those of Black or White race spend a greater proportion of their leisure time watching TV compared to those of Asian or Hispanic/Latino descent.^4^ The race and sex-stratified analyses (Table 3) may be underpowered given smaller samples in each subgroup. There is also the possibility of unmeasured confounders, even after adjusting for potential confounders including physical activity, age, race, sex, education, family history, smoking status, alcohol, and BMI (which could be a proxy for nutritional status). Although dietary intake could be a partial mediator for the association between television and CVD events, it could also be a confounder. Our study has the unique strength of featuring repeated measures of television viewing and CVD outcomes collected across a multi-decade follow-up period, in a national US sample, starting in young adulthood.

## CONCLUSION

In conclusion, higher television viewing levels in young adulthood and annual increases in television viewing into midlife are each significantly and independently associated with incident premature CVD events before age 60. Young adulthood represents an important window for early intervention and a time in which individuals establish television viewing behaviors for the rest of adulthood. However, irrespective of television viewing levels in young adulthood, decreasing television viewing levels from young adulthood to midlife may also lower the risk of premature CVD events.

## Supplementary Information

Below is the link to the electronic supplementary material.

Supplementary file1 (DOCX 368 KB)

## Data Availability

Data from the Coronary Artery Risk Development in Young Adults Study may be accessed through a manuscript proposal or ancillary study proposal (details at https://www.cardia.dopm.uab.edu/invitation-to-new-investigators).
