# Supplementary material for: Television Viewing from Young Adulthood to Middle Age and Premature Cardiovascular Disease Events: A Prospective Cohort Study
Source: J Gen Intern Med. 2024 Aug 22;39(14):2780–7. doi: 10.1007/s11606-024-08951-z (PMC11534955; doi:10.1007/s11606-024-08951-z)
Supplement: Supplementary file 1 — Supplementary file1 (DOCX 368 KB) [file 11606_2024_8951_MOESM1_ESM.docx]

Supplemental Figure A. Coronary heart disease (CHD) incidence, by race and sex

**
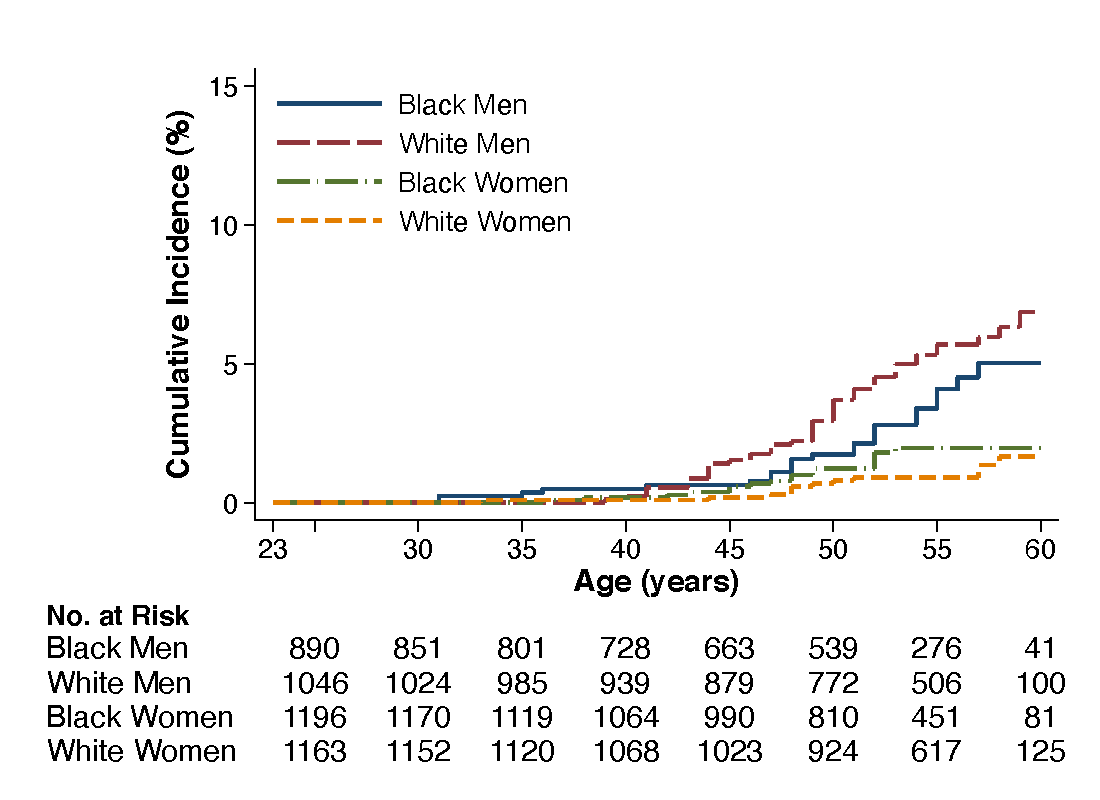
**

Supplemental Figure B. Congestive heart failure (CHF) incidence, by race and sex

**
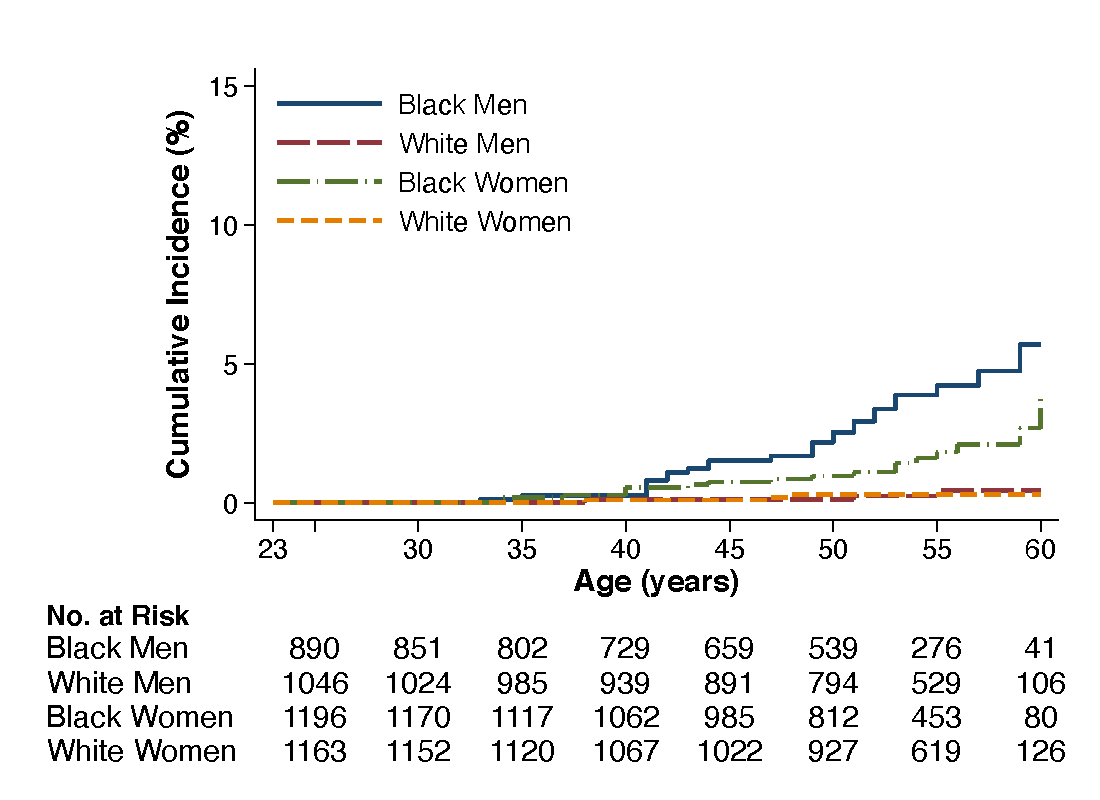
**

Supplemental Figure C. Stroke incidence, by race and sex

**
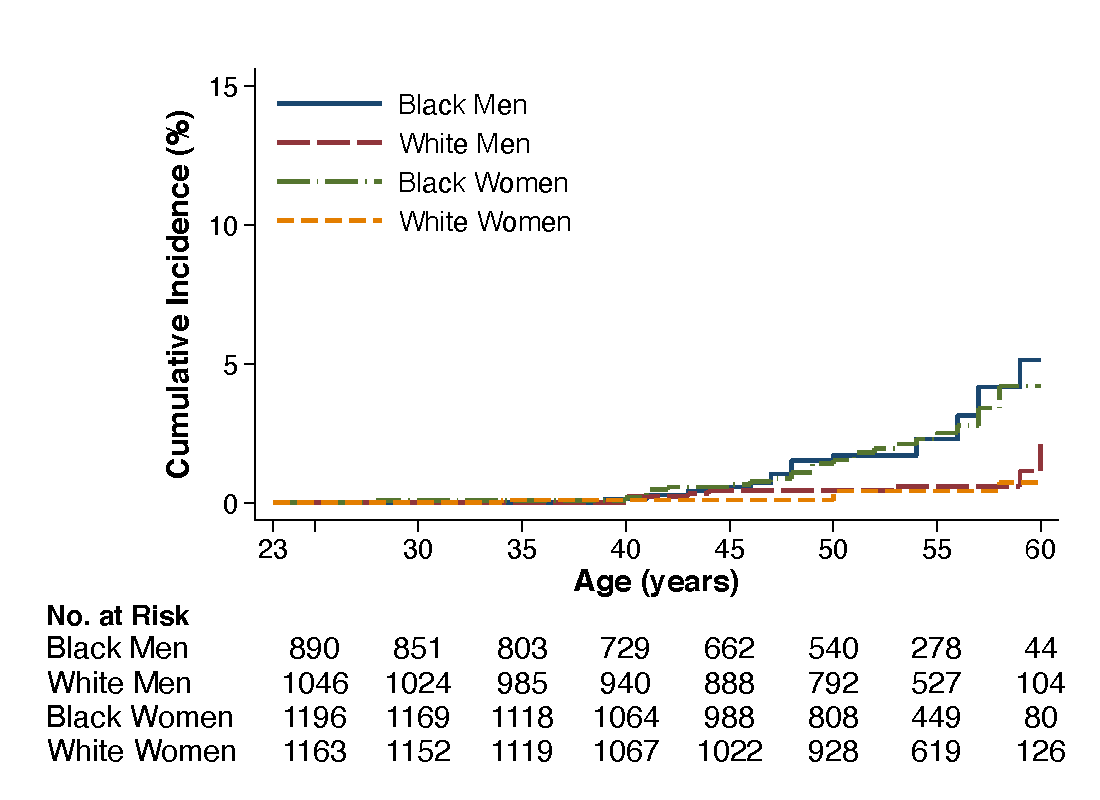
**

Supplemental Figure D. Cardiovascular disease incidence, by race and sex

**
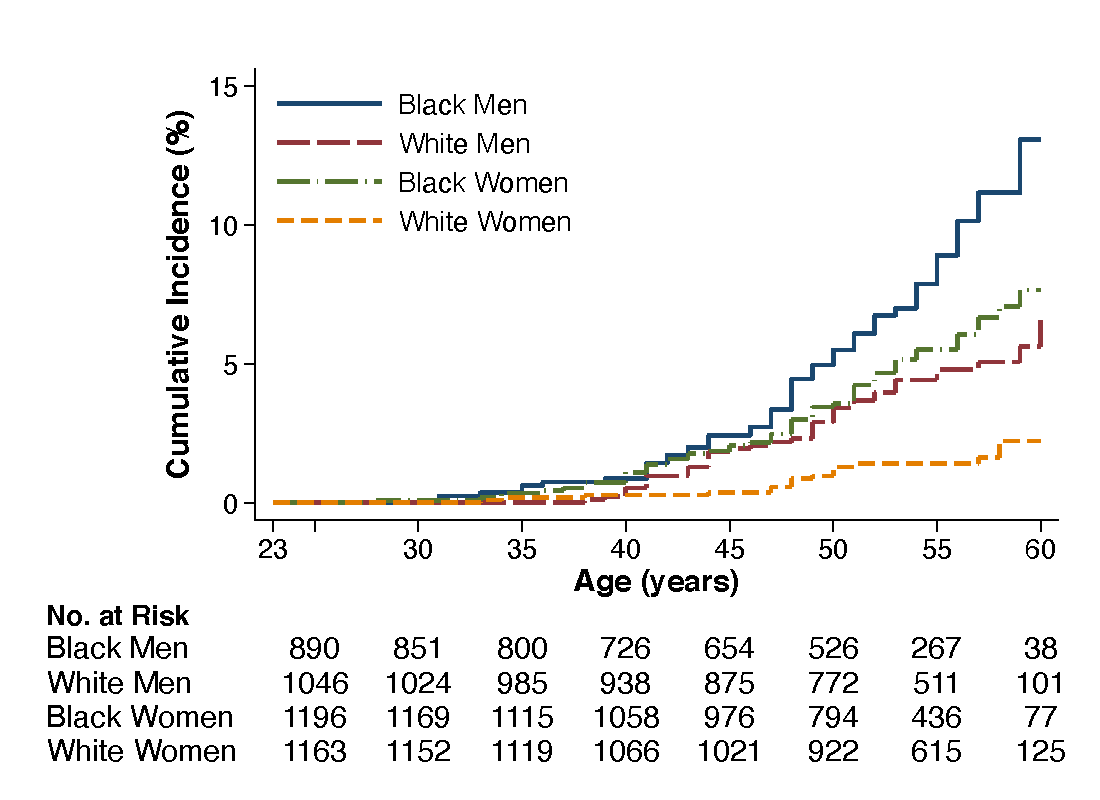
**
